# The Ontogeny of Sexual Size Dimorphism of a Moth: When Do Males and Females Grow Apart?

**DOI:** 10.1371/journal.pone.0106548

**Published:** 2014-09-03

**Authors:** R. Craig Stillwell, Andrew Daws, Goggy Davidowitz

**Affiliations:** 1 Department of Ecology and Evolution, University of Lausanne, Lausanne, Switzerland; 2 Department of Entomology, University of Arizona, Tucson, Arizona, United States of America; Oxford Brookes University, United Kingdom

## Abstract

Sexual dimorphism in body size (sexual size dimorphism) is common in many species. The sources of selection that generate the independent evolution of adult male and female size have been investigated extensively by evolutionary biologists, but how and when females and males grow apart during ontogeny is poorly understood. Here we use the hawkmoth, *Manduca sexta*, to examine when sexual size dimorphism arises by measuring body mass every day during development. We further investigated whether environmental variables influence the ontogeny of sexual size dimorphism by raising moths on three different diet qualities (poor, medium and high). We found that size dimorphism arose during early larval development on the highest quality food treatment but it arose late in larval development when raised on the medium quality food. This female-biased dimorphism (females larger) increased substantially from the pupal-to-adult stage in both treatments, a pattern that appears to be common in Lepidopterans. Although dimorphism appeared in a few stages when individuals were raised on the poorest quality diet, it did not persist such that male and female adults were the same size. This demonstrates that the environmental conditions that insects are raised in can affect the growth trajectories of males and females differently and thus when dimorphism arises or disappears during development. We conclude that the development of sexual size dimorphism in *M. sexta* occurs during larval development and continues to accumulate during the pupal/adult stages, and that environmental variables such as diet quality can influence patterns of dimorphism in adults.

## Introduction

Sexual differences in body size (sexual size dimorphism: SSD) are common in organisms and have attracted considerable interest in evolutionary biology for over a century [1], [2], [3]. Sexual size dimorphism varies considerably across all taxonomic levels; the degree and direction of dimorphism may vary substantially among populations within species, among species and among the major animal groups such as birds, mammals and insects [1], [4]. For example, females are often the larger sex in insects, whereas males are often the larger sex in mammals [4], [5]. This variation in size dimorphism is due to multiple sources of selection acting differentially on the sexes: fecundity selection for increased female size, sexual selection for increased male size and selection favoring small size in both sexes (through selection for short development time, [4]). Although numerous studies have focused on these evolutionary explanations for how selection can generate variation in dimorphism in adult body size, few studies have focused on how and when sexual size dimorphism arises during development [6], [7], [8], [9], [10]. Such studies are critical to understanding how size dimorphism evolves in adults because the proximate target of selection is the developmental process that determines growth and body size in immature stages [10].

There are three mutually non-exclusive ways for males and females to reach different sizes during growth and development: Males and females can differ in their size at hatching/birth, their growth rates and/or their development time [4]. Some studies have shown that the female-biased (females larger) dimorphism of insects is due to females growing faster than males, while other studies have shown that females prolong their growth and thus increase their size relative to males [10], [11], [12], [13]. Only a few studies have examined sex differences in size at hatching, but the general impression is that there are no differences in hatching size between males and females in insects [12].

In general, little is known about when males and females start to diverge in body size during development [7], [14], [15]. The results of the few studies that have examined when dimorphism develops in invertebrates are not consistent; differences in size between males and females does not occur until late in development in some species [12], [16], [17], [18], [19], [20], [21] whereas studies with other species show that dimorphism is present early in development [14], [20], [22], [23], [24]. Most prior studies on the ontogeny of sexual size dimorphism are limited because only one or a few life stages were examined. Detailed studies that explore the ontogeny of size dimorphism from egg hatching to adult eclosion are needed to fully understand when sexual size dimorphism arises during growth and development.

Ecological and environmental variables might influence the growth and development of the sexes differently and this could affect when males and females diverge in size during ontogeny. For example, female insects frequently exhibit greater phenotypic plasticity in body mass than males, creating variation in sexual size dimorphism within species [4]. Most of this sex difference in plasticity in size is in response to variation in diet quality and quantity, although some studies have shown that developmental temperature can also create sex-specific plasticity in size [22], [25], [26]. Because diet quality/quantity is known to be important in controlling insect growth and development and because it generates substantial sex-specific plasticity in body size, it is possible that diet quality/quantity will affect the ontogeny of sexual size dimorphism. However, no study has investigated whether diet quality/quantity affects when males and females diverge in size during development.

In this study, we use the hawkmoth, *Manduca sexta*, as a model system to examine sexual size dimorphism in each stage of development to determine when sexual dimorphism starts to develop and to see whether the magnitude of this dimorphism changes over time. In addition, we investigate if diet quality influences the ontogeny of sexual size dimorphism. We raised moths from egg hatching to the last larval developmental stage on three different qualities of diet (100, 80 and 60%, see below), measuring the mass of individual larvae once daily. We then measured the mass of pupae and emerging adults to test whether the degree of larval sexual dimorphism changed in the pupal or adult stages. To our knowledge, this represents the most detailed examination of the development of size dimorphism of any insect, especially in response to an environmental manipulation.

## Materials and Methods

### Natural history of *Manduca sexta*

The hawkmoth, *Manduca sexta* (Linnaeus), is a large moth (forewing length is ∼51–56 mm) distributed from South America to southern Canada [27], [28]. Adults lay eggs singly on the undersides of leaves of its host plants. The eggs hatch and larvae feed on the foliage for ∼20 days before burrowing into the soil to pupate [29]. Adult feeding does affect egg production in *M. sexta*, so it is not solely dependent on larval resources to make eggs [30]. As a result, body size of females at eclosion does not necessarily predict egg production.

### Experimental design

**Figure 1:**
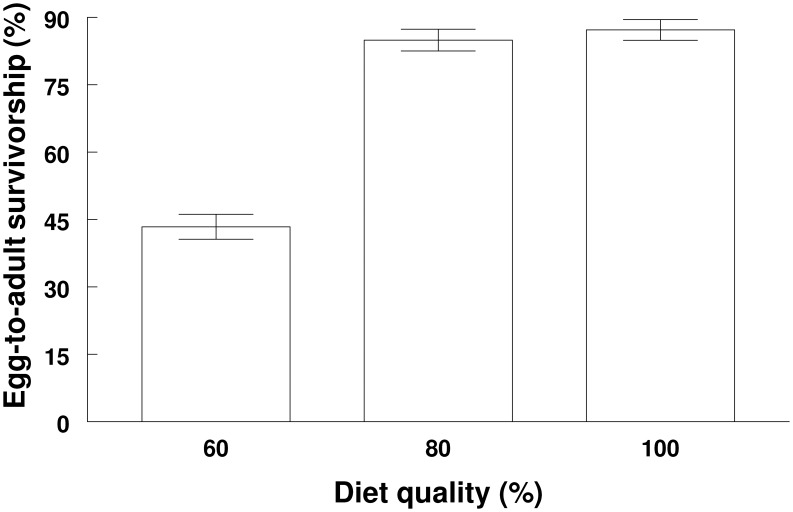
Egg-to-adult survivorship (%) (a) of *M. sexta* raised on three different diet qualities (60, 80 and 100%) (mean ± SEM).

The *M. sexta* population used in this study was outcrossed from laboratory colonies from Duke University, the University of Arizona and the University of Washington. We raised individual caterpillars from egg hatching to adult on three different diet qualities (100, 80 and 60%; all at 25°C; 16∶8, L:D) [31], [32] to examine when sexual dimorphism develops and whether environmental conditions can alter the ontogeny of dimorphism. The nutrients in the diet quality treatments were reduced by the appropriate proportions. For example, the 60% diet contained 60% of the nutrients per gram compared to the standard 100% diet (the standard diet used to raise the laboratory colonies). A non-nutritive bulk (Alphacel, ICN, Aurora, OH, USA) was added to the diet to make up the remaining portion, such that the quantity of food that larvae received in each treatment was identical. The vitamin, antibacterial and antifungal components were the same for all three diets. The formula for these diets can be found in [31]. Finally, we raised a similar number of larvae in the 100% (212 larvae) and 80% (219 larvae) diet treatments since mortality was similar in these treatments, but we raised ∼45–50% more larvae in the 60% treatment (318 larvae) due to substantial increase in mortality on this diet (Fig. 1).

Eggs were collected from the stock colony within 24 hours of being laid and were randomly divided among the three diet treatments. Eggs were placed into rectangular metal pans with the appropriate diet treatment (hatching larvae were supplied with ample food and were keep at low densities to minimize larval competition). Larvae were collected within 24 hours of hatching, weighed on an electronic balance and placed into individual plastic cups (Solo© P100, approximately 30 cm^3^ volume) with approximately 1 cm^3^ of their respective diet treatment. We weighed larvae daily until they reached the fifth instar (determined by when the larvae shed their head capsules), at which point larvae were transferred to larger plastic cups (Solo TP9, approximately 266 cm^3^ in volume) with approximately 16 cm^3^ of the appropriate diet. Fifth instar larvae were weighed daily until they reached their peak larval mass and secreted ecdysone, determined by the deposition of pink ommochrome pigments along the dorsum, the exposure of the dorsal vessel and the onset of wandering behavior [33]. We placed the larvae into 4×30 cm wooden blocks, drilled with ten 2.5 cm diameter holes to mimic the underground pupation chamber. The individuals in these blocks were kept at 25°C (16∶8, L:D) to pupate. One week later, the pupae were removed, sexed and weighed on an electronic balance. These pupae were transferred to plastic cups (Solo TP9, approximately 266 cm^3^ in volume). When the pupae turned black (indicating imminent eclosion), they were removed and placed into bakery bags. Eclosed adult moths were collected daily, weighed on an electronic balance and were placed into a –20°C freezer (to measure sex-specific allocation of resources for a separate study).

In total, we raised 502 individuals from egg to adult. For all analyses (except mortality analyses) on the ontogeny of size dimorphism, we used only individuals that survived to adults.

### Statistical analysis

**Figure 2:**
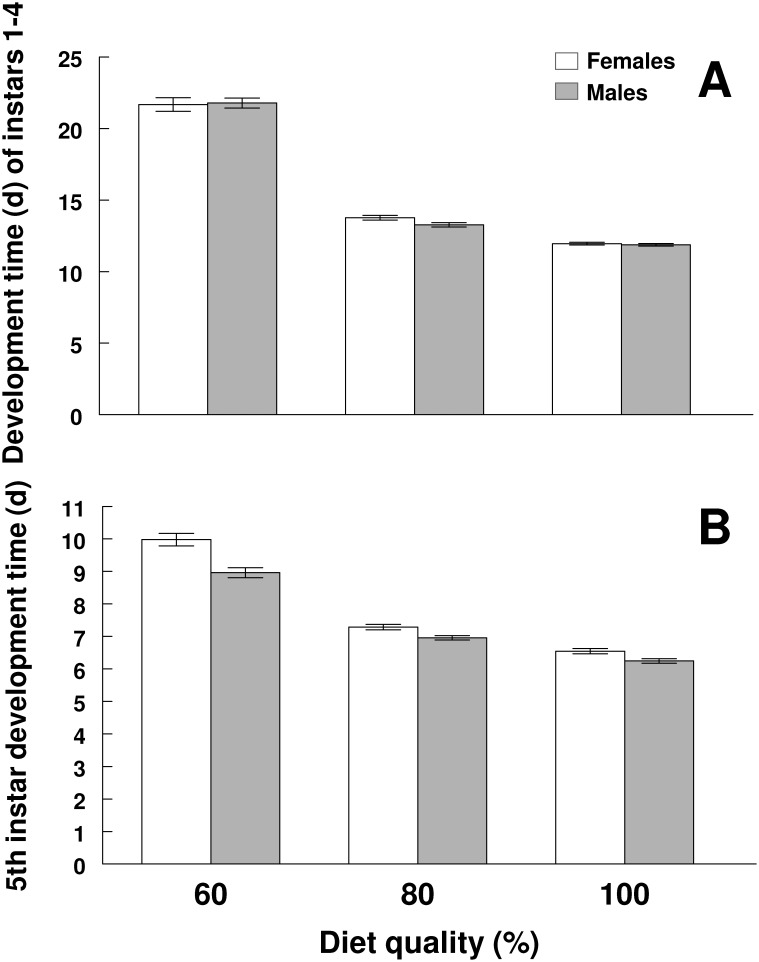
Development time of the 1^st^–4^th^ instar larvae (a) of *M. sexta* raised on three different diet qualities (60, 80 and 100%) (mean ± SEM). Development time of 5^th^ instar larvae (b) raised on the three different diet treatments.

We used logistic regression analysis (SAS PROC GENMOD) to determine the effect of diet quality on survivorship. Normal probability plots of all mass data revealed the data were approximately normally distributed. One inherent problem with the structure of our dataset is that there is variation in development time among individuals and among treatments (Fig. 2). This makes comparisons based on individual days problematic, especially among treatments since the larvae are not the same physiological age. We therefore adjusted for variation in development time by comparing the mass of larva at 25%, 50% and 75% of the development time from hatching to pupation, calculated separately for each individual larva. We also used a one-way ANOVA to examine when the sexes diverged in size within treatments and a two-way ANOVA to examine when the sexes diverge in size across treatments and whether this changed with the diet treatment.

Because we were interested in testing for a differential response in body size between males and females to our diet manipulation, we tested for sex-by-diet interactions in the ANOVAs. However, using interactions in ANOVA to test for changes in the magnitude of sexual size dimorphism can provide misleading results because the *proportional* or *relative* effects are of primary interest; ANOVA uses the *linear* difference between treatment means to test for interactions between factors [34], [35], [36]. Essentially, a scaling problem is created when there is a large effect of one variable on the overall mean. For example, increasing diet quality causes a large increase in body size such that the degree of dimorphism changes as a result of body size increasing [34]. We thus took a two-step to our analysis. For the main effects, we present the results based on our untransformed data. However, for all interactions, we present the results from log transformed data to resolve the scaling problem.

## Results

### Survivorship

As expected, 85% and 87% of larvae survived to the adult stage when raised on the 80% and 100% diet treatments, respectively, but only 43% of larvae made it to the adult stage when raised on the 60% diet treatment (χ^2^_2_ = 157, *P*<0.0001; Fig. 1).

### Development time

As expected, the development time of 1–4^th^ instar larvae decreased substantially with increasing diet quality (*F*_2,485_ = 1017, *P*<0.0001; Fig. 2A). However, there was no difference between sexes in development time (*F*_1,485_ = 0.78, *P* = 0.38) and this did not change with the type of diet they were raised on (*F*_2,485_ = 0.97, *P* = 0.38; Fig. 2A). Likewise, development time of 5^th^ instar declined considerably with increasing diet quality (*F*_2,485_ = 419, *P*<0.0001; Fig. 2B). The development time of 5^th^ instar females was considerably longer than in males (*F*_1,483_ = 41.0, *P*<0.0001; Fig. 2B), but this effect depended on the quality of diet the larvae were raised on; females had about a 5% longer development time than males when raised on the 100% and 80% diet treatments while females had about a 11% longer development time when raised on the 60% diet treatments (*F*_2,483_ = 6.60, *P* = 0.0015; Fig. 2B).

### Body mass – Within treatments

**Figure 3:**
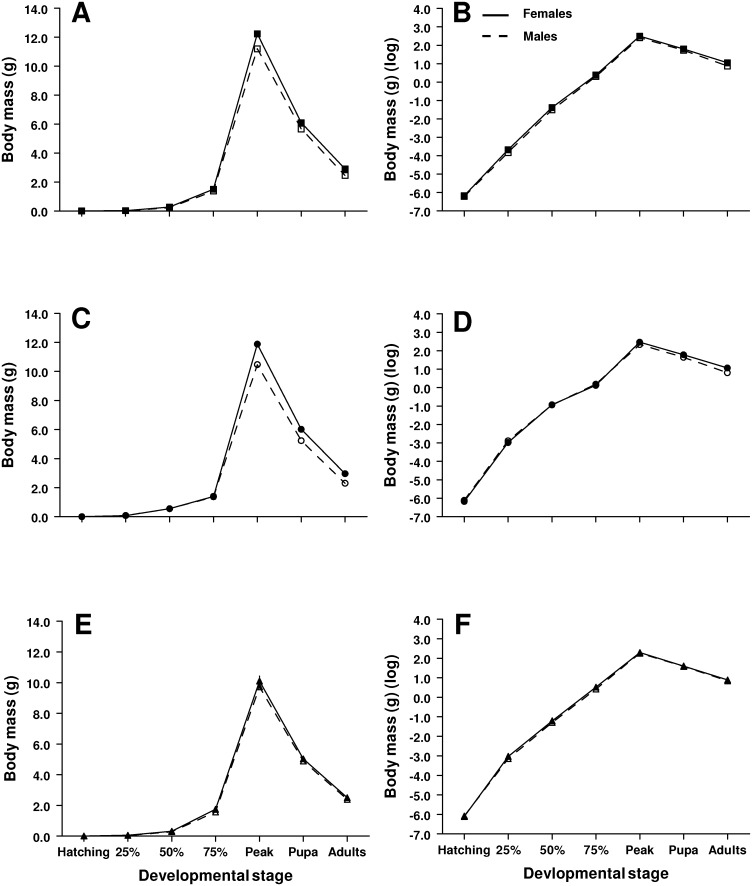
Mass of female and male larvae (a,c,e) of *M. sexta* raised on three different diet qualities: 100 (a,b), 80 (c,d) and 60% (e,f). Standard error bars are included, but are smaller than the symbols for some experimental treatments. Body mass of female and male larvae raised on the various diet treatments after log transformation (b,d,f). 25%, 50% and 75% represent body mass at 25%, 50% and 75% of the development time from hatching to pupation.

**Figure 4:**
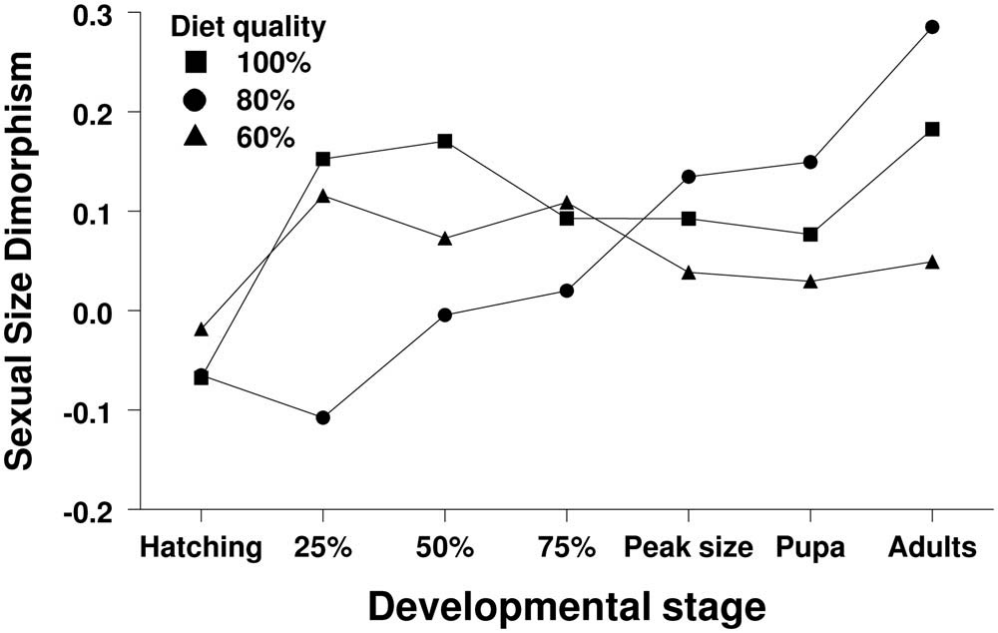
Sexual size dimorphism of moths raised on different qualities of diet. To estimate the magnitude of dimorphism for each developmental stage, we used the Lovich and Gibbons index, in which SSD = (size of the larger sex/size of the smaller sex)–1, made positive when females are the larger sex and negative when males are the larger sex [44].

Sexual size dimorphism first appeared at 25% of the development time from hatching to pupation when individuals were raised on the 100% diet treatment; females were about 15% larger than males during this stage (*F*_1,180_ = 6.35, *P = *0.01; Fig. 3A,4). This effect persisted all the way to the adult stage (*F*≥6.35, *P*≤0.01 for all stages; Fig. 3A,4), during which dimorphism became the most pronounced; females were about 18% larger than males during this stage (*F*_1,180_ = 28.8, *P<*0.0001; Fig. 3A,4). Males were about 10% larger than females at 25% of the development time from hatching to pupation when raised on the 80% diet treatment (*F*_1,179_ = 5.19, *P = *0.02; Fig. 3C,4) but this effect disappeared during the next few developmental stages (*F*≤0.38, *P*≥0.54; Fig. 3C,4). Females were about 13% larger than males at peak larval size (*F*_1,179_ = 52.6, *P<*0.0001; Fig. 3C,4) and this effect persisted to the pupal stage where females where about 15% larger than males (*F*_1,179_ = 56.0, *P<*0.0001; Fig. 3C,4). As in the 100% diet treatment, the dimorphism in the 80% diet treatment became the most pronounced in the adult stage; adult females were about 29% larger than adult males (*F*_1,179_ = 64.0, *P<*0.0001; Fig. 3C,4). Females were about 12% larger than males at 25% of the development time from hatching to pupation when raised on the 60% diet treatment, but the effect was not significant (*F*_1,121_ = 3.19, *P = *0.08; Fig. 3E,4) and disappeared in the next developmental stage (*F*_1,121_ = 1.17, *P = *0.28; Fig. 3E,4). Females were 11% larger than males at 75% of development time from hatching to pupation (*F*_1,121_ = 4.35, *P = *0.03; Fig. 3E,4) but the effect disappeared from all subsequent stages (*F*≤1.10, *P*≥0.30; Fig. 3E,4).

### Body mass – Across treatments

Body size increased with increasing diet quality at all stages of development as we expected (*F*≥5.88, *P*≤0.003 for all stages; Fig. 3). We also found that females became larger than males at 75% of the development time from hatching to pupation and that this effect persisted throughout development and into the adult stage (*F*≥5.92, *P*≤0.02; Fig. 3,4). In addition, we detected a marginally significant sex-by-diet interaction at hatching (*F*_2,480_ = 3.27, *P = *0.04) but the effect was not present in the next few developmental stages (*F*≤2.11, *P*≥0.11 for the next three stages). However, females were considerably larger than males at peak larval size in two of our three experimental treatments (100% and 80%) but not in the 60% diet treatment (sex-by-diet interaction: *F*_2,480_ = 4.44, *P = *0.01; Fig. 3,4). This pattern of female-biased dimorphism in the 100% and 80% diet treatments but not in the 60% diet treatment persisted to the pupal (*F*_2,480_ = 7.12, *P = *0.0009; Fig. 3,4) and adult stages (*F*_2,480_ = 8.25, *P = *0.0003; Fig. 3,4).

## Discussion

Most studies on sexual size dimorphism focus on evolutionary explanations for how selection can generate variation in dimorphism of adults. However, the proximate mechanisms that generate dimorphism remain poorly understood [4], [10], [14]. In particular, little is known about when males and females diverge in body size during ontogeny and whether the divergence in size between sexes changes with the quality of diet that larvae are raised on [4], [14], [37]. Knowing when the sexes diverge in time is important in understanding the targets of selection on SSD. For example, if males and females diverge during the early larval stages, selection may act on parameters of growth such as growth rate, development time or number of instars [4], [37]. Divergence among the sexes during the last larval instar, when 90% of growth occurs [38] suggests selection acting on nutrient conversion efficiency or growth rate. However, should SSD occur in the pupal to adult stage, selection may act on resource use efficiency during metamorphosis or on osmoregulatory mechanisms in the pupa. Thus, knowing when SSD occurs can provide insight into the possible physiological mechanisms involved.

Here we showed that female-biased dimorphism (females larger) arose early during development when larvae were raised on the 100% diet treatment and persisted to the adult stage, during which the dimorphism became even more pronounced. Female-biased dimorphism arose at peak larval mass when individuals were raised on the 80% diet treatment. This also persisted to the adult stage, during which the degree of dimorphism increased further. However, although females were larger than males at a few points during larval development when raised on the 60% diet treatment, this effect did not persist such that male and female adults were the same size. Interestingly, the overall degree of female-biased dimorphism increased considerably in the adult stage for the 100% and 80% diet treatments because males lost more mass than females at eclosion, a pattern that is common in Lepidoptera (39). The significant sex-by-diet interactions we detected late in development confirm that when dimorphism arises or whether it arises at all depends on the quality of diet individuals are raised on.

The appearance of sexual size dimorphism during larval development, as we observed in the 100% and 80% diet treatments, has also been observed in other insects. For example, [14] showed that dimorphism appears early in larvae and accumulates throughout development in a predictable stepwise manner. Presumably a stair-step pattern of the accumulation of dimorphism occurs because insect growth is limited by the physiological inability to grow much within an instar such that extra mass has to be accumulated in different instars [14]. Likewise, size dimorphism arises early in the fruit fly, *Drosophila melanogaster*, and persists to the adult stage though the magnitude of this dimorphism diminishes slightly over time [40]. Dimorphism also arises early in development in some speices of Odonates but then disappears in adults [19]. However, this same study also showed that dimorphism arises late in development (appearing during later larval instars or during the adult stage) in other species of Odonates. One potential explanation for this inconsistency among species is that early divergence between sexes in body size occurs in species that have a fixed instar number between sexes (as is the case in lab strains of *M. sexta* and *D. melanogaster*), but does not when the number of instars differs between sexes [12], [14]. More detailed studies are clearly needed to see if the appearance of dimorphism during the larval stage is a general phenomenon in insects with a fixed number of instars between sexes vs. those that do not. Another possibility is the quality of diets differs among studies because, as we observed in this study, dimorphism arises later in development (80% diet treatment) or not at all (60% diet treatment) depending on the quality of diet that individuals are raised on.

The greater loss in mass in males vs. females between the termination of growth and adult eclosion in the 100% and 80% diet treatments seems to be an important mechanism in generating adult patterns in sexual size dimorphism in insects [39], [40], [41]. This is not surprising because we would expect sexual selection to play a large role in producing sex differences in body size and other traits in adults but not during development when we would expect sexual selection to play no role. However, it’s not clear what physiological mechanisms produce this sex difference in weight loss at eclosion. [39] found that dimorphism increased considerably from the pupal to the adult stages in many species of Lepidoptera and was due to males losing more water content than females at eclosion, possibly because females need more water to allocate to maturing eggs. The change in dimorphism we observed from the pupal to the adult stage was due to males losing more mass than females (both lost weight at eclosion), but it is not clear if this was because males lost more water than females. However, females of *D. melanogaster* lose considerably more mass than males after the cessation of growth, such that the dimorphism at peak larval size is nearly twice that of the adults [40]. It is unclear why males lose more mass than females after growth stops in Lepidopterans but females lose more mass than males in fruit flies. However, the magnitude of adult size dimorphism in insects seems to be strongly influenced during this period. Future studies should thus focus on why these differences exist between insect groups and investigate the specific developmental mechanisms that generate these patterns.

Our study also examined whether environmental variables such as diet quality could affect when sex differences in body size arise and the persistence of this dimorphism over time, which no prior study has investigated. Our results suggest that declining diet quality makes it more difficult for females to become larger than males because dimorphism arose late in development in the 80% diet treatment and even though it arose briefly it had completely disappeared near the end of development in the poorest quality diet treatment. One potential adaptive explanation for this pattern is that females would have to develop for a very long time in order to become larger than males, exposing them to predation or other sources of mortality, which would decrease their fitness. However, a possible physiological explanation for this pattern is that the lack of nutrition makes it impossible for females to reach their target body size, which is substantially larger than males since they are the larger sex in this species. Males may not be affected as much by the lack of nutrition because they have a smaller target body size to reach.

Prior studies have shown that sexual size dimorphism may develop due to the sexes having a different number of total instars [12], [14]. However, the primary objective of our study was to determine *when* males and females become different sizes during development and whether the timing of this divergence is the same across environmental treatments; the developmental and physiological mechanisms that determine *how* males and females become different sizes was addressed in our prior studies [10], [42]. Although not mentioned in our previously published work, males and females have the same number of instars in laboratory colonies of *M. sexta* and this does not change across environmental treatments [43]. Consequently, the female-biased dimorphism of *M. sexta* cannot be attributed to a difference in instar number between the sexes.

In summary, we showed that differences in body size of males and females of *M. sexta* first arise during early larval development when raised on the 100% diet treatment and during late larval development when raised on the 80% diet treatment. Interestingly, while female larvae were already larger than male larvae near the end of development, the magnitude of dimorphism increased substantially in the transition from the pupal to the adult stage when they were raised on the good quality diet treatments (100% and 80%). However, while female larvae were larger than male larvae at a few developmental stages when raised on the poor quality diet treatment (60%), this dimorphism disappeared during the last stages of development. Our study demonstrates that examining the development of size dimorphism during all stages of development and under different environmental conditions provides potential insights into the mechanisms that generate dimorphism in adults.
